# Wound Integrity of 2.0 mm Transconjunctival Single-Plane Sclerocorneal Incision: A Comparison between a Motorized Injector with/without Pause Time and a Manual Injector

**DOI:** 10.1155/2016/8129298

**Published:** 2016-02-17

**Authors:** Hisanori Imai, Ayaka Fujii, Emiko Tani, Atsushi Azumi

**Affiliations:** ^1^Department of Ophthalmology, Kobe Kaisei Hospital, 3-11-15 Shinohara Kitamachi, Nada-ku, Kobe 657-0068, Japan; ^2^Department of Organ Therapeutics, Division of Ophthalmology, Kobe University Graduate School of Medicine, 7-5-2 Kusunoki-cho, Chuo-ku, Kobe 650-0017, Japan; ^3^Tani Eye Clinic, 3-1-7 Morigo-cho, Nada-ku, Kobe 657-0028, Japan

## Abstract

*Purpose*. To compare the final incision size and wound structure after the intraocular lens implantation from 2.0 mm transconjunctival single-plane sclerocorneal incision (TSSI) between the use of a motorized injector at first speed and the use of a manual injector.* Methods*. Patients were divided into three groups as follows: Group A, a manual injector, Group B, a motorized injector with 0.5 s pause time, and Group C, a motorized injector without pause time. The change in incision size and anterior segment optical coherence tomography findings of the wound structure were analyzed.* Results*. 110 eyes were enrolled (Group A: 40, Group B: 30, and Group C: 40). The averaged change in incision size (mm) was 0.08, 0.01, and 0.03 in Groups A, B, and C, respectively (*p* < 0.001). The incision enlargement in Group A was statistically larger compared with other groups (*p* < 0.01). Descemet's membrane detachments were seen in 26, 9, and 27 eyes one day after the surgery in Groups A, B, and C, respectively (*p* = 0.001). The rate of Descemet's membrane detachment in Group B was significantly lower than other groups (*p* < 0.01).* Conclusions*. The use of a motorized injector by fastest setting with 0.5 s pause time is the best for less wound damage in 2.0 mm TSSI.

## 1. Introduction

To date, the techniques for intraocular lens (IOL) implantation have been developed for the reduction of the incision size, because the smaller incisions can offer faster rehabilitation, lesser astigmatism, lesser inflammation, and fewer chances of wound leak and postoperative endophthalmitis after cataract surgery. Recently, the use of an injector system has become a standard technique, and various injector systems have been developed. Previous reports suggested that the construction and enlargement of the main incision are affected by the type of injector cartridges and the method of IOL implantation [1–5]. The motorized injector (AutoSert: Alcon Laboratories, Inc.) is one of the new injector systems for IOL implantation. The surgeon can customize the IOL insertion speed and set a pause time just before the IOL insertion by using this injector. Several reports have already suggested that faster IOL insertion speed can provide less wound enlargement compared to slower IOL insertion speed and that the use of it can offer less wound damage than the manual injector in clear corneal incisions (CCI) [6, 7]. On the other hand, the effects of the pause time on the wound damage have never been evaluated.

Many surgeons prefer CCI for cataract surgery [8] because of the ease of creation, absence of bleeding, and increased accessibility to the anterior chamber through the incision. On the other hand, the instability of CCI in the early postoperative period, the lack of conjunctival coverage over the incision, and a suspected role in postoperative endophthalmitis are still concerned [9–13]. Sugai, et al. reported the transconjunctival single-plane sclerocorneal incision as a new technique which can achieve merits of both CCI and sclerocorneal incisions [14].

In this study, we compared the final incision size and wound integrity after IOL implantation from 2.0 mm transconjunctival single-plane sclerocorneal incision between the use of a motorized injector with pause time, the use of a motorized injector without pause time, and the use of a manual injector.

## 2. Subjects and Methods

We performed prospective, randomized study at Kobe Kaisei Hospital. Our study was performed under the writing informed consent from each patient, the approval of the institutional review board in Kobe Kaisei Hospital, and the Tenets of the Declaration of Helsinki. Patients' criteria included surgeries performed at Kobe Kaisei Hospital from November 2013 through March 2014. No eye had ocular pathology other than cataract, and no eye had history of ocular surgery. All patients had performed phacoemulsification and received hydrophobic acrylic aspheric intraocular lens (AcrySof SN60WF IOL, Alcon Laboratories, Inc.) implantation with a D cartridge (Alcon Laboratories, Inc.) from 2.0 mm transconjunctival single-plane sclerocorneal incision by a single surgeon. Patients were randomly divided into three groups using an envelope method: Group A, where a manual injector (Alcon Monarch III injector: Alcon Laboratories, Inc.) was used for IOL implantation, Group B, where a motorized injector (AutoSert: Alcon Laboratories, Inc.) at fast speed (4.4 mm/s) with 0.5 s pause time was used for IOL implantation, and Group C, where a motorized injector (AutoSert: Alcon Laboratories, Inc.) at fast speed (4.4 mm/s) was used for IOL implantation. Pause time was not set for this group. Incision width was measured before and immediately after IOL implantation in each group using incision gauge (Tsuneoka microincision caliper: ASICO). Patients in whom the incision size enlarged by more than 2.0 mm after irrigation-aspiration and before IOL was implanted were excluded from the study. The following variables were analyzed: sex, eye, age, preoperative endothelial cell density, grade of nuclear sclerosis, IOL power, the change in wound size during the surgery, surgical induced astigmatism (SIA) 1 day and 1 week after the surgery, and anterior segment optical coherence tomography (ASOCT) findings of the wound structure 1 day and 1 week after the surgery. ASOCT was done for all the patients for main 2.0 mm incision using the SPECTRALIS Anterior Segment Module Optical Coherence Tomography (Heidelberg Engineering GmbH) and analyzed as reported previously [15].

### 2.1. Statistical Methods

We used Kruskal-Wallis *H* test followed by post hoc analysis using Mann-Whitney *U* test with Bonferroni correction to examine differences in age, endothelial cell density, IOL power, SIA, and the incision enlargement among each group. The chi-square test and Fisher's direct probability test and residual analysis were also used to compare the differences in sex, eye, grade of nuclear sclerosis, and ASOCT findings among each group. Statistical analyses were performed using statistical software (MedCalc version 12.7.5.0; MedCalc Software, Mariakerke, Belgium). Statistical significance was inferred for *p* < 0.05.

### 2.2. Surgical Procedures

All surgeries were performed by a single experienced surgeon (HI) in the same operative room and by the same phacoemulsification machine (Infiniti Vision System: Alcon Laboratories, Inc.). A side port incision was created with a 20G MVR blade (MVR-Lance: Mani, Inc.) after sub-Tennon's anesthesia was performed. Both dispersive and cohesive viscoelastic materials were injected to fill the anterior chamber and circular continuous capsulorhexis was performed using 23-gauge micro capsulorhexis forceps (Eye technology, Inc.) from the side port. A 2.0 mm bent transconjunctival single-plane sclerocorneal incision was made with a 2.0 mm slit knife (Alcon Laboratories, Inc.). Cortical hydrodissection was done and nucleus was emulsified using divide and conquer technique. Phacoemulsification was done using torsional phacoemulsification with a 0.9 mm Kelman mini-flare tip (Alcon Laboratories, Inc) and UltraSleeve (Alcon Laboratories, Inc). Irrigation-aspiration of cortical material was done using intrepid silicone-sleeve coaxial system (Alcon Laboratories, Inc). The incision width was measured after filling the anterior chamber and the bag with a cohesive viscoelastic materials using incision gauge (Tsuneoka microincision caliper: ASICO). Hydrophobic acrylic aspheric intraocular lens (AcrySof SN60WF IOL, Alcon Laboratories, Inc.) implantation was done using a D cartridge with a manual injector in Group A and a motorized injector in Groups B and C. Wound-assisted IOL insertion was done in each group. Final positioning of the IOL in bag was done using the lens hook from the side port. The incision width was again measured immediately after IOL implantation. The viscoelastic materials was removed using irrigation-aspiration. Finally, the anterior chamber was inflated by injecting a balanced salt solution through the side port incision to assess the integrity of the wound by digitally gauging intraocular pressure. No patient needed corneal stromal hydration of the incision.

## 3. Results

One hundred ten eyes were included in the study: 40 eyes in Group A, 30 eyes in Group B, and 40 eyes in Group C. The preoperative demographic data of the patients in each group were comparable (Table 1). The mean incision enlargement after IOL insertion was 0.08 mm, 0.01 mm, and 0.03 mm in Groups A, B, and C, respectively (*p* < 0.001, Kruskal-Wallis *H* test). The incision enlargements in Groups B and C were statistically significantly smaller than that of Group A (*p* < 0.01, Mann-Whitney *U* test with Bonferroni correction) (Table 2). The mean SIA 1 day after the surgery was 0.44 D, 0.53 D, and 0.47 D in Groups A, B, and C, respectively (*p* = 0.57, Kruskal-Wallis *H* test). The mean SIA 1 week after the surgery was 0.39 D, 0.30 D, and 0.38 D in Groups A, B, and C, respectively (*p* = 0.40, Kruskal-Wallis *H* test) (Table 2). One day after the surgery, the detachment of Descemet's membrane were seen in 26 eyes (65%), 9 eyes (30%), and 27 eyes (68%) in Groups A, B, and C, respectively (*p* = 0.001, chi-square test). The rate of Descemet's membrane detachment in Group B was significantly lower compared to that of Groups A and C (*p* < 0.01, residual analysis). The endothelial gap was seen in 13 eyes (33%), 8 eyes (27%), and 13 eyes (33%) in Groups A, B, and C, respectively (*p* = 0.95, chi-square test). The wound bulge was seen in 7 eyes (18%), 0 eyes (0%), and 5 eyes (13%) in Groups A, B, and C, respectively (*p* = 0.15, Fisher's exact probability test) (Table 3). One week after the surgery, the detachment of Descemet's membrane was seen in 11 eyes (28%), 4 eyes (13%), and 14 eyes (35%) in Groups A, B, and C, respectively (*p* = 0.21, Fisher's exact probability test). The endothelial gap was seen in 9 eyes (23%), 5 eyes (17%), and 10 eyes (25%) in Groups A, B, and C, respectively (*p* = 0.85, chi-square test). The wound bulge was seen in 1 eye (3%), 0 eyes (0%), and 0 eyes (0%) in Groups A, B, and C, respectively (*p* = 0.86, Fisher's exact probability test) (Table 3).

## 4. Discussion

As shown in Results, there were statistically significantly less incision enlargements after IOL implantation through 2.0 mm transconjunctival single-plane sclerocorneal incision using a motorized injector compared with a manual injector in the current study. Allen et al. reported that the incision enlargement after IOL implantation through 2.0 mm CCI was 0.08 mm and was 0.15 mm by a motorized injector with the fastest injection speed (4.4 mm/min) and a manual injector, respectively, and the difference was statistically significant [7]. Their results and ours are highly comparable even though the method of the incision is different between these reports. These results indicate that the use of a motorized injector can offer less wound stretch regardless of the type of incision and may support the positive use of the motorized injector even in case of transconjunctival single plane sclerocorneal incision for less incision enlargement.

In our study, IOL implantation using a motorized injector with 0.5 s pause time could decrease the rate of Descemet's membrane detachment statistically significantly compared with that using a motorized injector without pause time and a manual injector (30% versus 68% versus 65%). On the other hand, the rate of Descemet's membrane detachment using a motorized injector without pause was not statistically significant compared with that using a manual injector (68% versus 65%). As previously shown, Descemet's membrane detachment is potentially a serious complication of intraocular surgery [16] and can occur by several reasons, like the mechanical damage by reexpanding IOL in the incision [1], lower intraocular pressure just after the surgery [17], and impairment of the local endothelial pump mechanism [18]. We believe our result might be affected by the difference in the frequency of the mechanical damage by reexpanding IOL among groups because we adjusted the intraocular pressure at the end of the surgery for all patients and no patient needed corneal stromal hydration of the incision. Previous report suggested that slow IOL insertion affects clear corneal wound structure more than fast IOL insertion [15], so there is no doubt that the faster IOL injection is better for the less incision damage if the IOL injector could be held in the correct alignment throughout the process. On the other hand, if we could not hold the IOL in the correct alignment during the injection, the mechanical damage of the endothelial site of incision by reexpanding IOL in the incision can happen, especially when IOL was inserted using wound-assisted method. It is possible that the settling of the motorized injector of 4.4 mm/s without pause time may be fast enough to decrease the wound stretch but too fast to adjust the IOL alignment during the injection and that 0.5 s pause time may be enough to adjust the IOL in the correct alignment during the injection.

Previous report indicated that the rate of endothelial gap was 50% and 20% using a motorized injector (2.2 mm/s with 0.5 s pause time) and a manual injector through 2.2 mm CCI with stromal hydration, respectively, which was statistically different [6]. Some report indicated that the eye with endothelial gap had inappropriate intraocular pressure and improper incision angle just after the surgery [19–22] and predisposed these eyes for endophthalmitis [23]. The stromal hydration of CCI is often performed to help the sealing [24–26], and Fine et al. [22] and Calladine and Tanner [18] reported that the stromal hydration diminished the endothelial gap rate in CCI. From these results, we can expect that the integrity of CCI after IOL implantation using a manual injector is significantly less than that using a motorized injector even after the stromal hydration. In the current study, no difference could be elicited among groups with respect to endothelial gap after the surgery. We used a transconjunctival single-plane sclerocorneal incision which has superiority in the wound sealing compared with CCI [14] and did not need any stromal hydration for all patients. We expect that the wound integrity after the transconjunctival single-plane sclerocorneal incision is good enough because this incision method is a form of sclerocorneal incision, which does not usually require stromal hydration, and is the reason why the rate of endothelial gap was not affected by the difference of IOL implantation method.

The current study has several limitations. First, we did not compare the wound structure after IOL implantation by different pause time settings. Other pause time settings may offer less impact on the incision. We need further examination to find the best pause time setting. Second, we did not perform the comparison of the incision enlargement and wound integrity between transconjunctival single-plane sclerocorneal incision and CCI after IOL implantation using a motorized injector. These should be evaluated in future study.

In conclusion, we compared the wound integrity after IOL implantation between a motorized injector and a manual injector using transconjunctival single-plane sclerocorneal incision. We believe that the use of a motorized injector by fastest setting with pause time is the best method to decrease the incision enlargement and the wound damage in 2.0 mm transconjunctival single-plane sclerocorneal incision.

## Figures and Tables

**Table 1:** Preoperative demographic data for the patients.

	Manual (*n* = 40)	Motorized +0.5 s pause (*n* = 30)	Motorized (*n* = 40)	*p* value
Sex, men/women	20/20	14/16	11/29	0.09
Eye, right/left	25/15	15/15	25/15	0.49
Age (years), mean ± SD	73.0 ± 7.8	72.0 ± 12.2	77.1 ± 7.8	0.08
Endothelial cell density (cells/mm^2^), mean ± SD	2658.1 ± 354.4	2672.1 ± 450.6	2536.1 ± 310.9	0.49
NS grade (eyes), 0/1/2/3/4/5	0/3/31/1/4/1	1/0/28/0/1/0	0/1/32/5/1/1	0.78
IOL power (diopter), mean ± SD	19.5 ± 4.2	19.3 ± 4.7	21.0 ± 3.5	0.21

NS, nuclear sclerosis; IOL, intraocular lens; SD, standard deviation.

**Table 2:** Postoperative demographic data for the patients.

	Manual (*n* = 40)	Motorized +0.5 s pause (*n* = 30)	Motorized (*n* = 40)	*p* value
Incision enlargement (mm), mean ± SD	0.08 ± 0.06	0.01 ± 0.03^*∗∗∗*^	0.03 ± 0.05^*∗∗*^	6.38 × 10^−5^
Surgically induced astigmatism (diopter), mean ± SD				
After 1 day	0.44 ± 0.24	0.53 ± 0.24	0.47 ± 0.27	0.57
After 1 week	0.39 ± 0.24	0.30 ± 0.16	0.38 ± 0.21	0.40

^*∗∗*^ *p* < 0.01 and ^*∗∗∗*^ *p* < 0.001, Mann-Whitney *U* test with Bonferroni correction.

**Table 3:** Postoperative optical coherence tomography findings of the wound structure.

	Manual (*n* = 40)	Motorized +0.5 s pause (*n* = 30)	Motorized (*n* = 40)	*p* value
After 1 day				
Without damage	7	16^*∗∗*^	7	0.003
Detachment of Descemet's membrane	26	9^*∗∗*^	27	0.001
Endothelial gap	13	8	13	0.95
Bulge	7	0	5	0.15
After 1 week				
Without damage	23	22	20	0.14
Detachment of Descemet's membrane	11	4	14	0.21
Endothelial gap	9	5	10	0.85
Bulge	1	0	0	0.86

^*∗∗*^ *p* < 0.01, residual analysis.
